# Global Clones of *Escherichia coli* CTX-M-15/ST10 and CTX-M-65/ ST683 Isolated from Brazilian Recreational Freshwater

**DOI:** 10.1007/s00284-026-04790-9

**Published:** 2026-02-21

**Authors:** Renata Gaino, Amanda Haisi, João P. Araújo Jr., Angela Guillen, Fábio P. Sellera, Marcos B. Heinemann, Natália C. Gaeta

**Affiliations:** 1 Programa de Pós-Graduação em Saúde Única, Universidade de Santo Amaro, São Paulo, Brazil; 2 Faculdade de Medicina Veterinária e Zootecnia, Universidade de São Paulo, São Paulo, Brazil; 3 Universidade Estadual Paulista, Botucatu, Brazil; 4 Instituto de Estudos Avançados, São Paulo, Brazil; 5 Faculdade de Medicina Veterinária, Universidade Metropolitana de Santos, Santos, Brazil

## Abstract

The emergence of antimicrobial resistance (AMR) poses a critical global health threat, underscoring the interconnectedness of human, animal, and environmental health within the One Health framework. This study describes two multidrug-resistant (MDR) extended-spectrum beta-lactamase (ESBL)-producing *Escherichia coli* strains, classified as critical priority bacteria by the World Health Organization, identified in recreational freshwater in Brazil. A cross-sectional study was conducted to investigate the presence of ESBL-producing *Enterobacterales* in water samples from a river in a touristic Brazilian city, including the waterfall, stream, and areas outside the city center. Bacteria were cultured and isolated on MacConkey agar supplemented with ceftriaxone, identified by MALDI-TOF, and antimicrobial susceptibility testing was performed by disk diffusion. ESBL-producing strains were then subjected to whole-genome sequencing and bioinformatic analysis. Two ESBL-producing *E. coli* strains were recovered from the waterfall (USP-MG-W) and steam (USP-MG-B). These strains showed an MDR profile, with resistance to β-lactams, tetracyclines, aminoglycosides, quinolones and phenicols, but remained susceptible to carbapenems. USP-MG-B and USP-MG-W harbor the ESBL genes *bla*_CTX-M-15_ and *bla*_CTX-M-65_, respectively, highlighting their broad-spectrum cephalosporin resistance and clinical relevance. Predicted heavy metal resistance genes suggest AMR co-selection. USP-MG-B belongs to pandemic *E. coli* sequence type (ST) 10, closely related to Brazilian human isolates. At the same time, USP-MG-W is part of clonal complex 155 (ST683), associated with international livestock and poultry strains. Remarkably, this study is the first report of *E. coli* ST10 in Brazilian rivers and ST683 with *bla*_CTX−M−65_ in Brazil. The detection of ESBL clones in freshwater underscores the role of the environment as a reservoir of critical priority pathogens. These findings highlight the urgent need for a One Health approach to the AMR crisis and for strengthening the epidemiological surveillance of ESBL-producing Enterobacterales beyond human settings.

**Supplementary Information:**

The online version contains supplementary material available at 10.1007/s00284-026-04790-9.

## Introduction

Antimicrobial resistance (AMR) is a global health crisis assessed and mitigated using the One Health approach [1]. The AMR phenomenon is not confined to clinical settings but extends into natural environments [2], where resistant bacteria can persist and proliferate in soil, rivers, and oceans. These environments act as reservoirs and conduits for resistant bacteria, facilitating their transmission between humans, animals, and other ecosystems [3].

Freshwater is particularly significant in AMR because it can accumulate and disseminate antimicrobial-resistant bacteria from various sources, including wastewater discharge, agricultural runoff, and direct contamination from human and animal activities [4]. In aquatic environments, bacteria can share resistance genes through horizontal gene transfer, a mechanism that pollutants and antibiotics can exacerbate. This mechanism establishes a breeding ground for resistant bacteria, which can then be transported downstream and into larger water bodies, such as seas and oceans, increasing the risk of exposure to humans and wildlife [5].

Third- and fourth-generation cephalosporin-resistant *Enterobacterales*, such as the extended-spectrum beta-lactamase (ESBL)-producing *Escherichia coli*, are particularly concerning due to their frequent multidrug-resistant (MDR) profiles and ability to cause life-threatening [6]. Because of their clinical impacts and global public health burden, ESBL-producing *Enterobacterales* have been designated as critical priority pathogens by the World Health Organization (WHO), highlighting the need for surveillance and control measures [6].

Although ESBL-positive *E. coli* were predominantly found in healthcare settings, recent evidence indicates that these bacteria can spread rapidly across various hosts and environmental niches, including lakes [7], oceans, and recreational waters [8]. In Brazil, despite the country’s rich biodiversity and the widespread use of these environments for tourism and leisure, the occurrence of ESBL-producing bacteria in freshwater for recreational purposes has not been extensively studied.

The lack of robust data on Brazilian recreational waters as ESBL reservoirs underscores the need for comprehensive monitoring and management strategies that account for the interconnectedness of human, animal, and environmental health, in line with the One Health approach, to combat AMR effectively. Herein, we report the occurrence and genomic features of two host-generalist *E. coli* strains, belonging to the pandemic sequence type (ST) ST10 and the widespread ST683, isolated from a steam source and a waterfall, respectively.

## Materials and Methods

In July 2022, a surveillance study investigated critical priority ESBL-positive *Enterobacterales* in recreational freshwater (Rio Verde River) in Caldas, southern Minas Gerais, Brazil (21°55’23’’S, 46°23’15’’W) (Supplementary Fig. 1). The river runs through rural and urban areas with dairy farms, food production, residences, and direct wastewater discharge. Ethics approval was not required, as no live humans or animals were involved. Still, the strains were registered in the Sistema Nacional de National System for the Management of Genetic Heritage and Associated Traditional Knowledge (SisGen) under #A2695FE.

Three water samples (500 mL) were collected from different sites: a waterfall (near dairy farms), a stream (near residences), and a site downstream from a bathhouse and swimming pool. Surface water samples were collected in sterile polystyrene bottles, transported to 4 °C, and processed within 1 day. Brazilian regulations do not require permits for environmental sampling, but biosafety protocols were followed. Each sample (100 mL) was centrifuged (10,000 g, 30 min), and pellets were resuspended in three milliliters of Brain Heart Infusion broth and incubated (35 ± 2 °C, 24 h).

### Isolation, Identification, and Antimicrobial Susceptibility Testing

To detect the presence of ESBL-producing *Enterobacterales*, aliquots (10 µL) were streaked on MacConkey agar with ceftriaxone (2.0 µg/mL) [9]. Species were identified by MALDI-TOF MS. Antimicrobial susceptibility was tested using the disk-diffusion method [10], with 15 antibiotics (DME, Brazil) across multiple classes (disk concentration in µg): amoxicillin-clavulanate (10 µg), ampicillin (10 µg), aztreonam (30 µg), cefepime (30 µg), cefotaxime (30 µg), cefoxitin (30 µg), ceftazidime (30 µg), ceftriaxone (30 µg), ciprofloxacin (05 µg), doxycycline (30 µg), ertapenem (10 µg), gentamicin (10 µg), imipenem (10 µg), meropenem (10 µg), nalidixic acid (30 µg), norfloxacin (10 µg), sulfonamide-trimethoprim/sulfamethoxazole (1.25/23.75 µg), and tetracycline (30 µg). ESBL production was assessed using the double-disk synergy test [11]. Multidrug-resistant (MDR) isolates were defined as those resistant to at least three different drug classes [12].

### Genomic Analysis of ESBL-producing *E. Coli*

DNA was extracted (PureLink^®^ kit), quantified (Qubit™), and ~ 350 ng was used for library prep (Illumina COVIDSeq kit). Sequencing (2 × 300 bp) was performed on MiSeq. Quality control included FastQC (github.com/s-andrews/FastQC), TrimGalore (github.com/FelixKrueger/TrimGalore), and Trimmomatic (www.usadellab.org/cms/?page=trimmomatic). Assemblies were generated with Unicycler (github.com/rrwick/Unicycler) and annotated with Prokka (github.com/tseemann/prokka). Resistomes were identified via BacMet 2.0, MEGARes2.0, ResFinder, and CARD using Abricate (github.com/tseemann/abricate). Plasmids were analyzed using PlasmidFinder, mlplasmids (gitlab.com/sirarredondo/mlplasmids), and MOB-suite (github.com/phac-nml/mob-suite#docker-image). MLST, FimH, and phylogroups were determined in silico (https://cge.food.dtu.dk/services; http://clermontyping.iame-research.center ). A ≥ 95% identity threshold was used for gene predictions.

### Phylogenetic Analysis

Core-genome phylogeny was built using *E. coli* ST10 and ST683 genomes from Enterobase (https://enterobase.warwick.ac.uk/; Supplementary Table 1) and isolate UMG-B-W. Genomes were selected based on MLST, metadata, ANI (> 99%, via FastANI, github.com/ParBLiSS/FastANI), completeness (≥ 90%), and contamination (≤ 5%) via CheckM (github.com/Ecogenomics/CheckM). ROARY (https://github.com/sanger-pathogens/Roary) generated core-genome alignments, and phylogenies were constructed in MEGA v11 (neighbor-joining, 1,000 bootstraps, Jukes-Cantor model).

## Results and Discussion

Only two ESBL-producing *Enterobacterales* isolates were recovered. Both strains were identified as *E. coli* by MALDI-TOF and subsequently confirmed by whole-genome sequencing (WGS). The ESBL-positive *E. coli* strains (USP-MG-W and USP-MG-B) were recovered from the waterfall and the stream, respectively.

The main phenotypic and genotypic results of both strains are presented in Table 1. Both isolates demonstrated phenotypic MDR to clinically significant drugs, such as aztreonam, ceftriaxone, cefoxitin, cefotaxime, cefepime, ceftazidime, sulfamethoxazole-trimethoprim, nalidixic acid, ciprofloxacin, gentamicin, and tetracycline. The USP-MG-B was also resistant to aztreonam. On the other hand, both strains remained susceptible to imipenem, meropenem, ertapenem, and amoxicillin-clavulanate. Here, we described MDR *E. coli* in freshwater in the Brazilian State of Minas Gerais, and interestingly, MDR non-coliform heterotrophic bacteria were previously detected in recreational water sources in the South region of the same State [13], indicating the dissemination of these microorganisms in these sites.

**Table 1:** Genomic data of *Escherichia coli* ST10 (USP-MG-B) and *Escherichia coli* ST683 (USP-MG-W) recovered from recreational water in Brazil

Characteristics	*E. coli* USP-MG-B	*E. coli* USP-MG-W
Source	River water - stream	River water - waterfall
ATB resistance profile^a^	ATM, CRO, CTX, CPM, CAZ, CFO, SUT, NAL, CIP, GEN, TET	ATM, CRO, CTX, CPM, CAZ, CFO, SUT, NAL, CIP, GEN, TET
Genome Size (bp)	4.599.265	4.690.065
No. of contigs	113	86
N50 (bp)	133,199	121,321
Sequence depth	87x	176x
No. of CDSs^b^	4260	4379
G + C content (%)	50.66	50.85
tRNAs (*n*)	58	59
rRNAs (*n*)	2	3
MLST (ST)^c^	ST10	ST683
cgMLST	120,736	88,575
Serogroup	H9:O101	H21:O30
Phylogroup	A	B1
FimH-type	H54	H121
Resistome		
β-lactams	*bla*_CTX−M−15_	*bla*_CTX−M−65_, *bla*_OXA−10_, *ARR-3*
Aminoglycosides	*–*	*ant(3’’)-Ia*,* aadA*
Tetracyclines	*tet(A)*	*–*
Quinolones	*–*	*qnrS1*
Phenicols	*–*	*cmlA1*
Heavy Metal	*arsBCR*,* cusABCF*,* nikBCDE*,* zntA*,* zraPRS*	*arsBCR*,* cusABCFS*,* nikBCDE*,* znuABC*,* zraPRS*
Virulome	*ecp*,* ent*,* fimACH*,* gsp*,* ibeB*	*cfaABCD*,* ent*,* fimACH*,* ecp*,* gsp*,* ibeB*
Plasmidome	*–*	IncR
Genbank Accession	JBHZTJ000000000	JBHZTK000000000

^a^ATB, antibiotic; CRO, ceftriaxone; CTX, cefotaxime; CPM: cefepime; ATM: aztreonam; NAL: nalidixic acid; CIP: ciprofloxacin; TET: tetracycline; GEN: gentamycin, SUT: sulfametoxazole-trimethoprim

^b^CDSs, coding sequences

^c^MLST, multilocus sequence type; S.T., sequence type

At the genomic level, USP-MG-B generated 2,694,856 paired-end reads, assembled into 113 contigs, with 81× coverage, a G + C content of 50.66%, and a genome size of 4,599,265 bp. Similarly, USP-MG-W generated 1,353,620 paired-end reads, assembled into 86 contigs, with a G + C content of 50.85% and a genome size of 4,690,065 bp.

Resistome analysis revealed the presence of genes encoding resistance to β-lactams (*bla*_CTX−M−15_) and tetracyclines (*tetA)* in the USP-MG-B strain. In contrast, genes encoding resistance to β-lactams (*bla*_CTX−M−65_ and *bla*_OXA−10_), aminoglycosides (*ant(3’’)-Ia*), quinolones (*qnrS1*), and phenicols (*cmlA1*) were detected in USP-MG-W. These genotypic findings corroborate the phenotypic resistance profiles observed in both strains. Additionally, in silico analysis revealed the presence of heavy metal tolerance genes in both strains, including arsenic (*arsBCR*), nickel (*nikBCDE*), zinc (*zraPRS*), and copper (*cusABCF*). Heavy metal tolerance genes, such as *zraPRS*, have been associated with intrinsic cephalosporin resistance [14]. Given the region’s mining activity, their presence may reflect local heavy metal exposure as well as co- or cross-selection with antibiotic resistance.

*E. coli* strains USP-MG-B and USP-MG-W belonged to phylogroups A and B1, respectively. The phylogroup B1 has been frequently associated with cattle [15]. Indeed, several dairy herds are located along the riverbank, and their residues can reach the water, which may explain the isolation of *bla*_CTX−M−65_-positive *E. coli*. Moreover, several important virulence genes were predicted in both strains, with *ibeB* and *ompA* being particularly noteworthy (Table 1). These genes are associated with *E. coli*’s ability to cross the blood-brain barrier and cause neonatal meningitis [16], raising concern due to potential human exposure to recreational waters.

A replicon of an IncR plasmid (named as IncR-MG-W) was in silico predicted in the USP-MG-W strain and harbors the *bla*_CTX−M−65_ gene, which was flanked downstream by an insertion sequence (IS) IS5 family transposase (Supplementary Fig. 3a). Resistance genes in USP-MG-W were associated with an IncR plasmid, known for its broad host range and carriage of multiple AMR genes despite lacking conjugation machinery. Notably, this plasmid has been found to carry genes conferring resistance to a wide array of antibiotics, including β-lactams, sulfonamides, quinolones, aminoglycosides, tetracyclines, chloramphenicol, and trimethoprim [17]. The present study identified multiple resistance genes within the IncR plasmid (Supplementary Fig. 3b). The *bla*_CTX−M−65_ gene in USP-MG-W was flanked by an IS5 transposase (Supplementary Fig. 3c), demonstrating the complexity of the genetic context surrounding both genes and indicating potential for mobilization.

USP-MG-B and USP-MG-W belong to serogroups H9:O101 and H21:O30, and sequence types ST10 and ST683, respectively. ST10 is a host-generalist pathogen widely distributed across the human-animal-environment interface, and its detection in environmental samples has been steadily increasing [18]. Specifically in Brazil, a recent nationwide genomic study revealed that this *E. coli* lineage has been present in the country since at least 1989, and is associated with ESBL, carbapenemase, and/or MCR production [18]. Although the presence of *E. coli* ST10 has been previously documented in urban-impacted coastal waters in Brazil [19], to our knowledge, this is the first report of its identification in a Brazilian river.

**Fig. 1:**
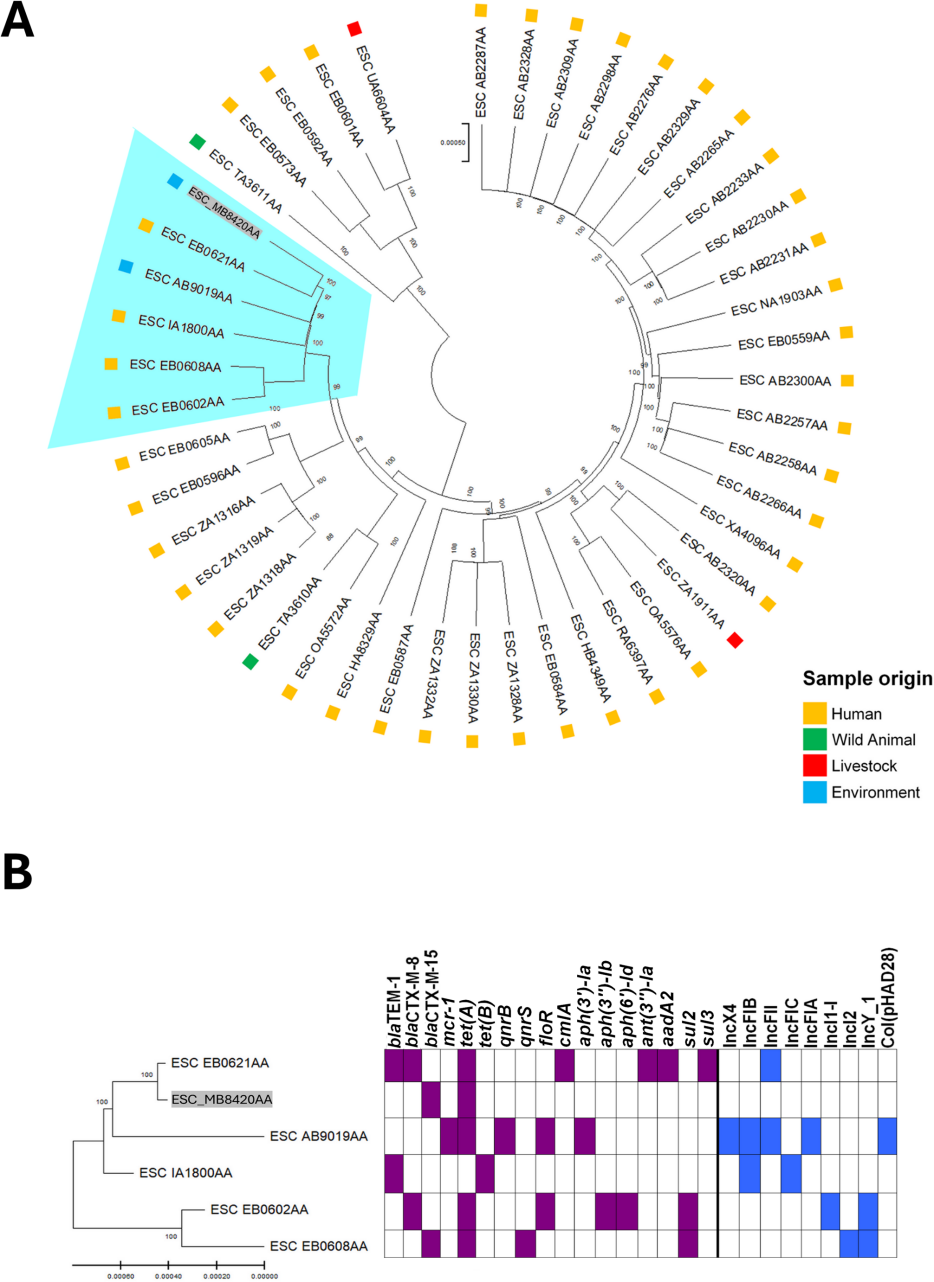
In **A**, a core-genome phylogenetic tree of 50 *E. coli* ST10 strains, highlighting the cluster with USP-MG-B (Enterobase accession # ESC_MB8420AA; gray). In **B**, a subtree cluster depicting the resistome and plasmidome of the isolates

Interestingly, in USP-MG-B, *bla*_CTX−M−15_ and *tet(A)* were in silico predicted as chromosomal. Moreover, the core-genome phylogenetic analysis for USP-MG-B focused exclusively on Brazilian strains, selecting 45 genomes to construct the final phylogenetic tree (Fig. 1). Most of these strains were isolated from human samples, revealing the spread of ST10 in this host in Brazil. USP-MG-B clustered with five other strains, primarily from humans and one from the environment (Fig. 1a), indicating a close relationship between a human and the environmental isolate, supported by pairwise distances of 99.9999% and 99.9998%, respectively. A comparison of the resistome and plasmidome within this cluster revealed discrepancies, and the *tet(A)* gene was present in most strains (Fig. 1b).

Similarly, the ST683 has been recovered from various hosts, including livestock and pigs [20] and humans [21]. Moreover, in Brazil, *E. coli* ST683, co-harboring *mcr-1* and *bla*_CTX−M−55_ genes, was isolated from touristic coastal water in the Northeastern region [22]. To our knowledge, no description of an ST683 strain carrying *bla*_CTX−M−65_ has been reported in the literature. Therefore, this study is the first to report an *E. coli* ST683 strain carrying *bla*_CTX−M−65_. The identification of *bla*_CTX−M−65_ in this lineage raises concerns about the potential for horizontal gene transfer and the emergence of novel resistant clones. This highlights the importance of continuous genomic surveillance to detect emerging resistance mechanisms and their dissemination across different bacterial populations.

**Fig. 2:**
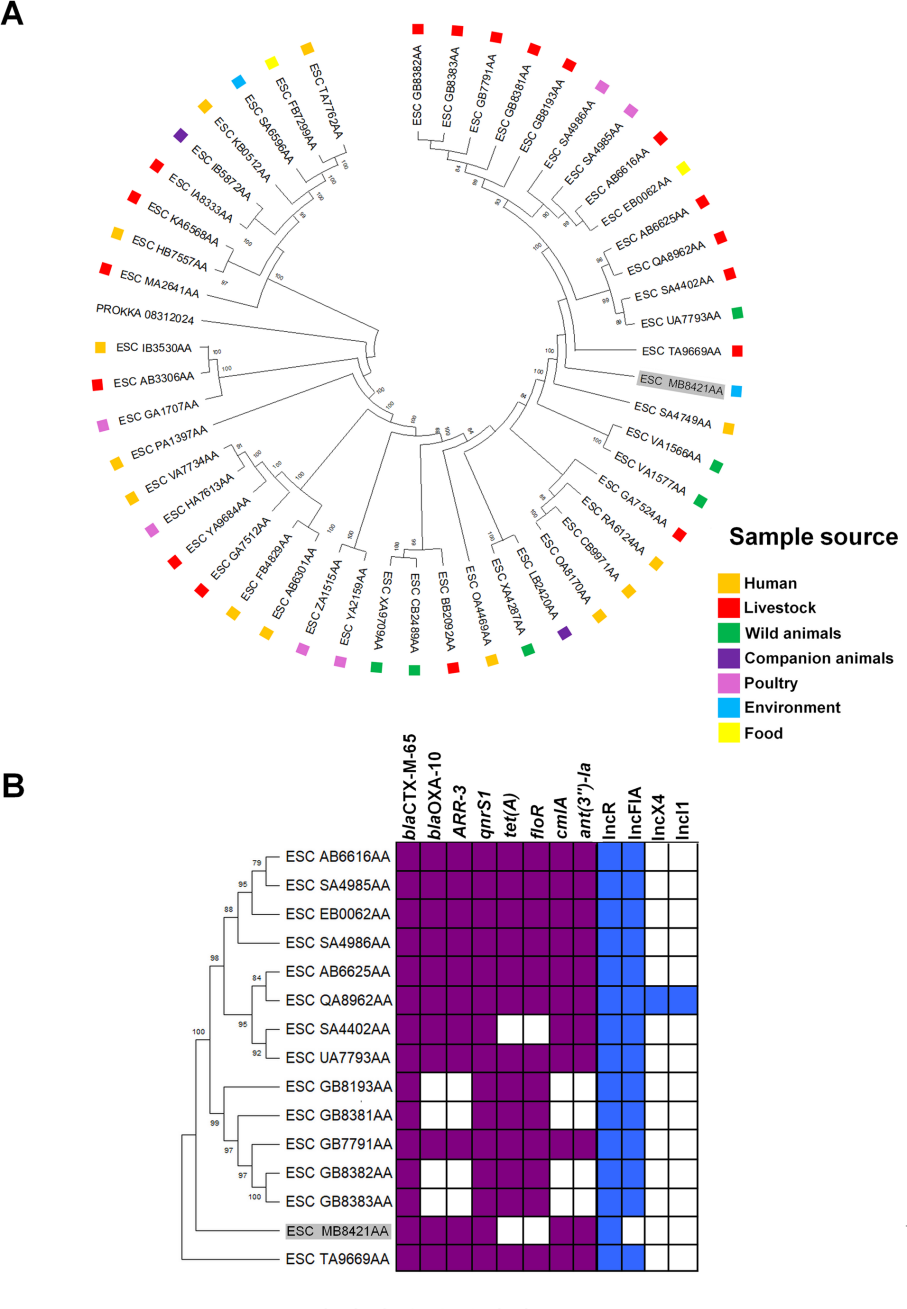
In **A**, the core-genome phylogenetic tree of 50 *E. coli* ST683 strains highlighting the USP-MG-W in gray (Enterobase accession # ESC_MB8421AA) and its similarity with other ST683 strains from livestock, human, food and wild animal sources. In **B**, a subtree shows the analogous resistome and plasmidome of the isolates of the USP-MG-W cluster

Furthermore, its core phylogenetic analysis included 49 isolates from 21 countries, including the USA, Canada, Estonia, Germany, Ireland, France, Switzerland, Nigeria, Australia, China, Gambia, Lithuania, Denmark, Thailand, Uganda, Tanzania, the U.K., Bangladesh, Japan, and Cambodia (Fig. 2). The results placed USP-MG-W within a clade like other ST683 isolates from livestock, poultry, wild animals, food, and humans from the USA, Canada, and Estonia (Fig. 2a), all carrying *bla*_CTX−M−65_ and *qnrS1* genes and harboring an IncR plasmid (Fig. 2b), reinforcing the One Health context of *E. coli* ST683.

ESBL-producing bacteria have been increasingly reported in several Brazilian freshwater environments in different regions, with the CTX-M group being the most frequently detected. In this regard, in the Southeastern region, a mercury-tolerant CTX-M-2-producing *E. coli* ST219 was recovered from the Doce River in Minas Gerais, which was previously used as a recreational site before the catastrophic mining dam disaster [23]. In São Paulo, the most populous state in Brazil, CTX-M-15-producing *E. coli* ST648 and ST617 were detected in a stream [24] and urban lake water [25], respectively. Additional reports from São Paulo include a CTX-M-14-producing *E. coli* ST354 isolated from a stream [26] and a CTX-M-2-producing *E. coli* ST1775 isolated from an urban stream [27]. In the South, CTX-M-15, CTX-M-3, CTX-M-2, and CTX-M-8-producing *E. coli* were isolated from urban superficial river water in Paraná [28]. In the North, CTX-M-15-producing *E. coli* ST471 was detected in an Amazonian Lake in Pará [29], and in the Northeast, CTX-M-producing *E. coli* ST131 was recovered from an urban lake in Bahia [30]. These studies highlight the environmental dissemination of CTX-M-type-producing *E. coli* in Brazil, with a predominance of those harboring CTX-M-15. Moreover, the presence of these WHO-critical-priority *E. coli* in freshwater environments appears to be closely associated with human-derived clones, including international high-risk lineages such as ST131, ST617, and ST648, underscoring the influence of anthropogenic pressure through contamination of rivers, streams, and other recreational water sources.

In our surveillance, the globally relevant *bla*_CTX−M−15_ was isolated from the steam. On the other hand, the *bla*_CTX−M−65_, part of the CTX-M-9 group (cluster 14), was first identified in 2008 in an *E. coli* strain isolated from a urine sample of an outpatient in the United States [31]. The *bla*_CTX−M−65_ gene is widely distributed. According to the data available in Enterobase (https://enterobase.warwick.ac.uk/), the gene is more frequent in Asia, followed by South America, Europe, North America, Oceania and Africa. Regarding the source of isolates, the *bla*_CTX−M−65_ gene is most commonly detected in humans, followed by the environment, poultry, livestock, companion animals, wild animals, food, wastewater, and pork. (Supplementary Fig. 2). In Brazil, the *bla*_CTX−M−65_ gene was previously detected in wildlife [32] and in *Salmonella* Infantis from a human patient [33]. To our knowledge, no environmental isolate harboring the *bla*_CTX−M−65_ gene has been detected to date; therefore, we report the first description of a *bla*_CTX−M−65_-positive *E. coli* from a Brazilian recreational freshwater body.

The Brazilian Environmental Sanitation Technology Company (CETESB) monitors freshwater coliforms but does not assess antimicrobial resistance genes or virulence factors [34]. This lack of genetic-level surveillance leaves a critical gap in public health data, underscoring the importance of studies like ours that investigate resistance mechanisms and pathogen genotypes in water sources. In this regard, a One Health approach should guide surveillance programs focused on recreational waters, agricultural runoff, and wastewater, enabling early detection of emerging AMR threats. Additionally, public awareness campaigns and collaboration across health, veterinary, and environmental sectors are crucial for mitigating the impact of AMR in aquatic environments.

Although our study provides valuable genomic and epidemiological data on WHO-critical-priority *E. coli*, due to logistical constraints and the exploratory nature of the work, only a limited number of water samples were processed. The inclusion of temporal replicates, measurements of physico-chemical water parameters, and quantification of microbial load would allow a more comprehensive assessment of the presence and spread of ESBL-positive isolates and facilitate the identification of additional clones. Therefore, our findings offer an initial snapshot of this environment while highlighting the need for future research incorporating multiple sampling points, measurements of environmental parameters, and microbial quantification to understand better the dissemination, dynamic patterns, and genetic traits of ESBL-producing *E. coli* circulating in this area.

## Conclusions

This study is the first to survey ESBL-producing bacteria in the Rio Verde River, specifically a recreational freshwater area, contributing to regional AMR surveillance. Detecting two clinically relevant MDR and CTX-M-positive *E. coli* strains, especially the first report of ST683 carrying *bla*_CTX−M−65,_ enhances global knowledge of ST10 and ST683 lineages. It provides a baseline for identifying AMR risk zones in this watershed. Finally, our findings highlight the need for a One Health perspective, as both isolates were associated with phylogroups linked to poultry and cattle and shared similar resistomes. Our results support the need for continuous monitoring of ESBL-producing bacteria in environmental sources to control the spread of these medically important pathogens emerging at the human-animal-environment interface.

## Supplementary Information

Below is the link to the electronic supplementary material.

Supplementary Material 1

Supplementary Material 2

Supplementary Material 3

Supplementary Material 4

## Data Availability

Sequence data is publicly available on Genbank by accession numbers JBHZTJ000000000 and JBHZTK000000000.
