# Supplementary material for: Global Clones of Escherichia coli CTX-M-15/ST10 and CTX-M-65/ ST683 Isolated from Brazilian Recreational Freshwater
Source: Curr Microbiol. 2026 Feb 21;83(4):205. doi: 10.1007/s00284-026-04790-9 (PMC12924868; doi:10.1007/s00284-026-04790-9)
Supplement: Supplementary file 2 — Supplementary Material 2 [file 284_2026_4790_MOESM2_ESM.docx]

**Global clones of *Escherichia coli* CTX-M-15/ST10 and CTX-M-65/ ST683 isolated from Brazilian recreational freshwater**

Renata Gaino^1^, Amanda Haisi^3^, João P. Araújo Júnior^3^, Angela Guillen^4^, Fábio P. Sellera^2,5^, Marcos B. Heinemann^2^, Natália C. Gaeta^1,2*^

^1^ Universidade de Santo Amaro, São Paulo, Brazil.

^2^ Faculdade de Medicina Veterinária e Zootecnia. Universidade de São Paulo, São Paulo, Brazil.

^3^ Universidade Estadual Paulista, Botucatu, Brazil.

^4^ Instituto de Estudos Avançados, São Paulo, Brazil.

^5^ Faculdade de Medicina Veterinária. Universidade Metropolitana de Santos, Santos, Brazil.

***Corresponding author**: [ngaeta@prof.unisa.br](mailto:ngaeta@prof.unisa.br).


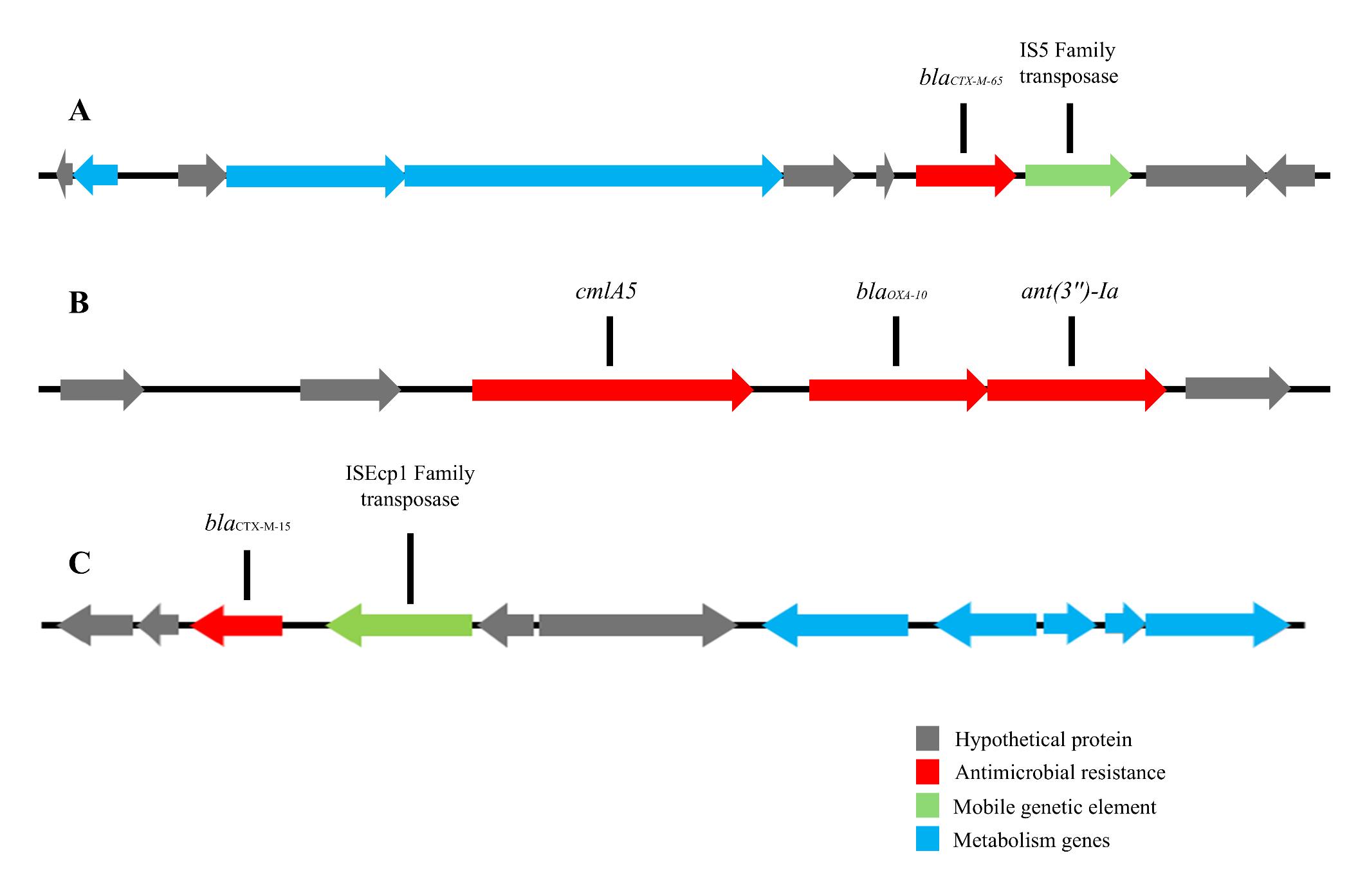
**Supplementary Fig. 3** Schematic figure showing the genetic context of the *bla*_CTX-M-65_ ESBL gene (a) and other important antimicrobial resistance genes (a) in the IncR plasmid of the environmental *E. coli* USP-MG-W ST683 strain, and (c) genetic context of the chromosomal *bla*_CTX-M-15_ in the environmental *E. coli* USP-MG-B ST10.
