# Supplementary material for: Global Clones of Escherichia coli CTX-M-15/ST10 and CTX-M-65/ ST683 Isolated from Brazilian Recreational Freshwater
Source: Curr Microbiol. 2026 Feb 21;83(4):205. doi: 10.1007/s00284-026-04790-9 (PMC12924868; doi:10.1007/s00284-026-04790-9)
Supplement: Supplementary file 4 — Supplementary Material 4 [file 284_2026_4790_MOESM4_ESM.docx]

**Global clones of *Escherichia coli* CTX-M-15/ST10 and CTX-M-65/ ST683 isolated from Brazilian recreational freshwater**

Renata Gaino^1^, Amanda Haisi^3^, João P. Araújo Júnior^3^, Angela Guillen^4^, Fábio P. Sellera^2,5^, Marcos B. Heinemann^2^, Natália C. Gaeta^1,2*^

^1^ Universidade de Santo Amaro, São Paulo, Brazil.

^2^ Faculdade de Medicina Veterinária e Zootecnia. Universidade de São Paulo, São Paulo, Brazil.

^3^ Universidade Estadual Paulista, Botucatu, Brazil.

^4^ Instituto de Estudos Avançados, São Paulo, Brazil.

^5^ Faculdade de Medicina Veterinária. Universidade Metropolitana de Santos, Santos, Brazil.

***Corresponding author**: [ngaeta@prof.unisa.br](mailto:ngaeta@prof.unisa.br).

**Supplementary Table 1.** Identification and epidemiological features of *E. coli* ST10 and ST683 strains used in the core-genome phylogenetic analysis.

| **Enterobase ID** | **Source** | **Collection Date** | **Location** | **ST** |
| --- | --- | --- | --- | --- |
| ESC_NA1903AA | Human | 1999 | Brazil | 10 |
| ESC_XA4096AA | Human | 1999 | Brazil |  |
| ESC_AB2329AA | Human | 2009 | Brazil |  |
| ESC_AB2233AA | Human | 2012 | Brazil |  |
| ESC_CA2587AA | Human | 2013 | Brazil |  |
| ESC_AB2230AA | Human | 2013 | Brazil |  |
| ESC_AB2231AA | Human | 2013 | Brazil |  |
| ESC_AB2257AA | Human | 2013 | Brazil |  |
| ESC_AB2258AA | Human | 2013 | Brazil |  |
| ESC_AB2266AA | Human | 2013 | Brazil |  |
| ESC_AB2287AA | Human | 2013 | Brazil |  |
| ESC_AB2298AA | Human | 2013 | Brazil |  |
| ESC_AB2309AA | Human | 2013 | Brazil |  |
| ESC_AB2328AA | Human | 2013 | Brazil |  |
| ESC_AB2276AA | Human | 2014 | Brazil |  |
| ESC_AB2320AA | Human | 2014 | Brazil |  |
| ESC_AB2300AA | Human | 2015 | Brazil |  |
| ESC_OA5572AA | Human | 2016 | Brazil |  |
| ESC_OA5576AA | Human | 2016 | Brazil |  |
| ESC_AB2265AA | Human | 2016 | Brazil |  |
| ESC_RA6397AA | Human | 2017 | Brazil |  |
| ESC_TA3610AA | Wild Animal | 2019 | Brazil |  |
| ESC_ZA1328AA | Human | 2019 | Brazil |  |
| ESC_ZA1330AA | Human | 2019 | Brazil |  |
| ESC_ZA1332AA | Human | 2019 | Brazil |  |
| ESC_AB9019AA | Environment | 2020 | Brazil |  |
| ESC_MB5230AA | Food | 2022 | Brazil |  |
| ESC_HB4349AA | Urine | 2015 | Brazil |  |
| ESC_EB0559AA | Human | 2016 | Brazil |  |
| ESC_EB0573AA | Human | 2017 | Brazil |  |
| ESC_HA8329AA | Human | 2013 | Brazil |  |
| ESC_EB0596AA | Human | 2017 | Brazil |  |
| ESC_EB0608AA | Human | 2018 | Brazil |  |
| ESC_EB0584AA | Human | 2017 | Brazil |  |
| ESC_EB0605AA | Human | 2019 | Brazil |  |
| ESC_UA6604AA | Livestock | 2017 | Brazil |  |
| ESC_IA1800AA | Human | 1999 | Brazil |  |
| ESC_EB0621AA | Human | 2019 | Brazil |  |
| ESC_EB0592AA | Human | 2017 | Brazil |  |
| ESC_EB0587AA | Human | 2017 | Brazil |  |
| ESC_TA3611AA | Wild Animal | 2019 | Brazil |  |
| ESC_EB0602AA | Human | 2018 | Brazil |  |
| ESC_ZA1911AA | Livestock | 2018 | Brazil |  |
| ESC_EB0601AA | Human | 2018 | Brazil |  |
| ESC_ZA1316AA | Human | 2019 | Brazil |  |
| ESC_ZA1318AA | Human | 2019 | Brazil |  |
| ESC_ZA1319AA | Human | 2019 | Brazil |  |
| ESC_EB0602AA | Human | 2018 | Brazil |  |
| ESC_GB8382AA | Livestock | 2017 | United States | 683 |
| ESC_GB8383AA | Livestock | 2017 | United States | |
| ESC_GB7791AA | Livestock | 2017 | United States | |
| ESC_GB8381AA | Livestock | 2017 | United States | |
| ESC_GB8193AA | Livestock | 2017 | United States | |
| ESC_SA4986AA | Poultry | 2016 | Canada |  |
| ESC_SA4985AA | Poultry | 2016 | Canada |  |
| ESC_AB6616AA | Human | 2011 | France |  |
| ESC_EB0062AA | Food | 2017 | Canada |  |
| ESC_AB6625AA | Livestock | 2016 | Canada |  |
| ESC_QA8962AA | Livestock | 2015 | Canada |  |
| ESC_SA4402AA | Livestock | 2017 | United States | |
| ESC_UA7793AA | Wild Animal | 2018 | United States | |
| ESC_TA9669AA | Livestock | 2017 | Estonia |  |
| ESC_SA4749AA | Human | 2015 | Germany |  |
| ESC_VA1566AA | Wild Animal | 2016 | United States | |
| ESC_VA1577AA | Wild Animal | 2016 | United States | |
| ESC_GA7524AA | Livestock | 2015 | Ireland |  |
| ESC_RA6124AA | Human | 2013 | France |  |
| ESC_CB9971AA | Human | 2018 | Switzerland | |
| ESC_OA8170AA | Human | 2019 | Nigeria |  |
| ESC_LB2420AA | Companion Animal | 2023 | United States | |
| ESC_XA4287AA | Wild Animal | 2012 | Australia |  |
| ESC_OA4469AA | Human | 2017 | China |  |
| ESC_BB2092AA | Livestock | 2022 | United States | |
| ESC_CB2489AA | Wild Animal | 2019 | Gambia |  |
| ESC_XA9709AA | Wild Animal | 2019 | Gambia |  |
| ESC_YA2159AA | Poultry | 2019 | United States | |
| ESC_ZA1515AA | Poultry | 2022 | United States | |
| ESC_AB6301AA | Livestock | 2022 | United States | |
| ESC_FB4829AA | Human | 2019 | France |  |
| ESC_GA7512AA | Livestock | 2015 | Lithuania |  |
| ESC_YA9684AA | Livestock | 2021 | France |  |
| ESC_HA7613AA | Poultry | 2014 | Denmark |  |
| ESC_VA7734AA | Human | 2016 | France |  |
| ESC_PA1397AA | Human | 2015 | Thailand |  |
| ESC_GA1707AA | Poultry | 2016 | Uganda |  |
| ESC_AB3306AA | Livestock | 2022 | United States | |
| ESC_IB3530AA | Human | 2021 | Tanzania |  |
| ESC_MA2641AA | Livestock | 2019 | United States | |
| ESC_HB7557AA | Human | 2019 | United Kingdom | |
| ESC_KA6568AA | Livestock | 2018 | United States | |
| ESC_IA8333AA | Livestock | 2018 | United States | |
| ESC_IB5872AA | Companion Animal | 2023 | United States | |
| ESC_KB0512AA | Human | 2019 | Bangladesh | |
| ESC_SA6596AA | Environment | 2017 | Japan |  |
| ESC_FB7299AA | Food | 2022 | Cambodia | |
| ESC_TA7762AA | Human | 2018 | Denmark |  |
